# The Determination of Trimethylamine N-Oxide and Organic Acids Connected with Gut Microbiota in the Urine of Parkinson’s Disease Patients: A Pilot Study

**DOI:** 10.3390/ijms26104575

**Published:** 2025-05-10

**Authors:** Paulina Gątarek, Małgorzata Pawełczyk, Barbara Bobrowska-Korczaka, Joanna Giebułtowicz, Andrzej Głąbiński, Joanna Kałużna-Czaplińska

**Affiliations:** 1 Institute of General and Ecological Chemistry, Faculty of Chemistry, Lodz University of Technology, 116 Zeromskiego Street, 90-924 Lodz, Poland; 2 CONEM Poland Chemistry and Nutrition Research Group, Lodz University of Technology, 90-924 Lodz, Poland; 3 Department of Neurology and Stroke, Medical University of Lodz, 90-549 Lodz, Poland; malgorzata.pawelczyk@umed.lodz.pl (M.P.); andrzej.glabinski@umed.lodz.pl (A.G.); 4 Department of Toxicology and Food Science, Faculty of Pharmacy, Medical University of Warsaw, Banacha 1 Street, 02-097 Warsaw, Poland; barbara.bobrowska@wum.edu.pl; 5 Department of Drug Chemistry, Pharmaceutical and Biomedical Analysis, Faculty of Pharmacy, Medical University of Warsaw, Banacha 1, 02-097 Warsaw, Poland; joanna.giebultowicz@wum.edu.pl

**Keywords:** Parkinson’s disease, PD, metabolites, gut microbiota, disease progression, chromatographic techniques

## Abstract

Parkinson’s disease (PD) progression appears closely tied to gut microbiota alterations, with microbial metabolites potentially influencing neurodegeneration. This pilot study employed GC-MS and LC-MS/MS to analyze the urinary levels of gut-derived metabolites—including succinic acid, *p*-hydroxyphenylacetic acid, homovanillic acid (HVA), vanillylmandelic acid (VMA), adipic acid, and trimethylamine N-oxide (TMAO)—in 20 PD patients versus 20 age-matched controls. The key findings revealed that PD patients exhibited significantly elevated succinic acid (*p* = 0.0018) and HVA (*p* = 0.0002) levels alongside reduced TMAO (*p* = 1.65 × 10^−5^). Notably, succinic acid showed an inverse correlation with disease severity (Hoehn and Yahr scale: r = −0.63; *p* = 0.0028), while TMAO demonstrated a strong positive association (r = 0.81; *p* = 0.00001). The elevated HVA, a dopamine metabolite, may serve as a potential biomarker for monitoring levodopa treatment efficacy. These results suggest that gut microbiota metabolites contribute to PD pathogenesis, with TMAO and succinic acid emerging as promising biomarkers for tracking disease progression. This study highlights the clinical potential of non-invasive urinary metabolite profiling using GC-MS and LC-MS/MS techniques. However, further investigation through larger-scale studies is needed to confirm these findings and elucidate the underlying mechanisms connecting gut microbiota dysbiosis to PD neurodegeneration.

## 1. Introduction

Parkinson’s disease (PD) is a chronic and progressive neurodegenerative disorder that currently affects over 10 million people. The disease is clinically characterized by the loss of dopaminergic neurons in the substantia nigra, resulting in typical motor symptoms such as bradykinesia, rest tremor, rigidity, and postural instability [1]. In addition to these motor presentations, PD is most commonly linked with a wide range of non-motor symptoms including sleep disturbance, constipation, depression, fatigue, and cognitive impairment, all of which significantly reduce quality of life.

Currently, PD diagnosis is heavily dependent on the observation of motor symptoms, which only appear after 50–70% of dopaminergic neurons have already been lost [2,3,4]. This delay in diagnosis is a critical flaw in clinical practice because it excludes early intervention and neuroprotective strategies. Thus, the identification of early biomarkers is of crucial significance to improve disease detection and management [5]. The exact cause of the disease remains unknown. While in a minority of cases there is an involvement of genetic factors in PD, the majority of PD cases are idiopathic, suggesting the probable substantial role of environmental and lifestyle factors. Among these, interest in the involvement of gut microbiota has gained increasing attention [2,3,4]. Experimental evidence from animal models shows that the transplantation of PD patient gut microbiota into mice overexpressing α-Syn produces greater motor dysfunction compared to transplantation from controls [6]. This observation strongly supports the role of gut microbial community composition in influencing disease phenotypes and in the hypothesis that gut-derived entities, such as microbial metabolites, contribute causally towards PD pathogenesis.

The gut microbiota is defined by the holistic collection of microorganisms living in the gut, such as bacteria, viruses, fungi and other microorganisms. The microbiota plays a key role in numerous physiological processes like nutrient acquisition, immune system regulation, intestinal barrier integrity, and pathogen defense. Dysbiosis, or the imbalance of the microorganisms in gut microbial communities, has been implicated in numerous diseases—from metabolic diseases and autoimmune disorders to neurodegenerative diseases like PD. A growing number of studies are pointing to a link between the gut microbiota and PD, suggesting that changes in the microbiota may be both a cause and effect of neurodegenerative processes. A number of studies have demonstrated significant differences between the gut microbiota of PD patients and healthy individuals [1,7,8,9,10]. Moreover, there is evidence showing the occurrence of the inauthentic deposition of α-Syn in the enteric nervous system due to inflammation or gut microbial imbalances, and from there progress into the brain through the vagus nerve [11,12,13]. This concept is the basis of the gut–brain axis, a bidirectional communication network with neural, hormonal, and immunologic routes that link gut function and microbial metabolism to central nervous system (CNS) activity. Therefore, the role of changes in the gut microbiota has been postulated as an important element in the development of neurological dysfunctions leading to neurodegeneration. Many patients with PD experience gastrointestinal symptoms (e.g., constipation), many years before the onset of neurological symptoms. Changes in the composition and metabolism of the gut microbiota are also observed in PD individuals [1,14,15,16,17,18,19]. Metabolites of bacterial origin are extremely important mediators of communication between the gut microbiota and the brain, such as trimethylamine N-oxide (TMAO) and various organic acids—short-chain fatty acids (SCFAs), such as acetic acid, propanoic acid and butyric acid, but also branched chain fatty acids (BCFAs) like isovaleric acid, lactic acid and succinic acid, as well as other bacterial metabolites like hippuric acid and *p*-hydroxyphenylacetic acid.

TMAO is one interesting biomarker that may reflect the state of the gut microbiota. It is a metabolite formed mainly by the action of gut bacteria on dietary components containing methyl groups such as choline, betaine and carnitine. The role of TMAO in inflammatory and metabolic processes has been extensively studied, but its potential importance in the context of PD still remains largely unknown. It has been postulated that TMAO is associated with the severity and progression of PD. In PD, higher TMAO levels have been associated with disease severity, neuroinflammation, motor dysfunction, and the onset of dementia [20,21]. TMAO may act by augmenting systemic inflammation and by influencing the misfolding and aggregation of α-Syn, a critical pathogenic process in PD.

Another important metabolite linked to the gut microbiota is succinic acid, which is involved in the metabolism of the tricarboxylic acid (TCA) cycle. It is a metabolite derived from the intestinal microbiota, as a byproduct of certain bacteria and a major cross-feeding metabolite between gut microbes. It has many functions, including playing a key role in intestinal homeostasis, regulating nutrient metabolism, participating in energy metabolism or influencing susceptibility to pathogens and diseases associated with inflammation in the body [22]. An alteration in the succinate level can be a sign of disturbances to host–microbiome metabolic interactions that are responsible for inflammation and mitochondrial injury, both of which are significant features of PD pathology.

Interestingly, although *Roseburia intestinalis* does not directly produce homovanillic acid (HVA), it increases the amount of *Bifidobacterium longum* in the gut, which in turn is capable of producing this metabolite. This suggests that R. intestinalis may indirectly support HVA synthesis by stimulating the growth of B. longum. The gut–brain axis may play a key role in transducing the effects of changes in the microbiota on the neurotransmitter function in the CNS [23].

There are few studies in the scientific literature focusing on the analysis of TMAO and gut microbiota-linked organic acids in the urine of PD patients or linking these metabolites to disease severity. Urine is a valuable biofluid in metabolomics research since it can be accessed non-invasively, with high levels of metabolites, and can also represent systemic and gut-derived metabolic changes. Urine metabolomics has been a valuable tool for identifying disease-related biochemical signatures and potential biomarkers.

In PD, urinary metabolomics offers selective insight into host metabolism and microbial function. Changes in urinary metabolites can serve as indicators of changes in mitochondrial function, inflammation, neurotransmitter metabolism, and gut microbial communities. For example, the urinary excretion of TMAO, succinic acid, and HVA can indicate not only PD-associated neurochemical changes but also alterations in gut microbial communities and their metabolic end products.

Consequently, in this study, we aimed to investigate the urinary levels of some gut microbiota-derived metabolites like TMAO, succinic acid, *p*-hydroxyphenylacetic acid, HVA, vanillylmandelic acid (VMA), and adipic acid in patients with PD compared to healthy individuals. Using advanced analytical techniques such as gas chromatography–mass spectrometry (GC-MS) and liquid chromatography–tandem mass spectrometry (LC-MS/MS), we tried to establish the metabolic profile of PD and investigate potential correlations between metabolite levels and disease severity as assessed by the Hoehn and Yahr (H-Y) scale, marking a novel approach in this area of research.

To the best of our knowledge, this is the first study to integrate a comprehensive analysis of microbiota-derived metabolites in the urine of PD patients using both GC-MS and LC-MS/MS, which has enabled the identification of new potential non-invasive biomarkers—such as TMAO and succinic acid—associated with disease progression as assessed by the Hoehn and Yahr scale.

Through the identification of specific microbial metabolites associated with PD, the present study aimed to shed more light on the gut–brain axis in neurodegeneration and pave the way for the development of early diagnostic biomarkers as well as novel therapeutic strategies, such as dietary interventions and microbiota-guided therapies.

## 2. Results

**Figure 1:**
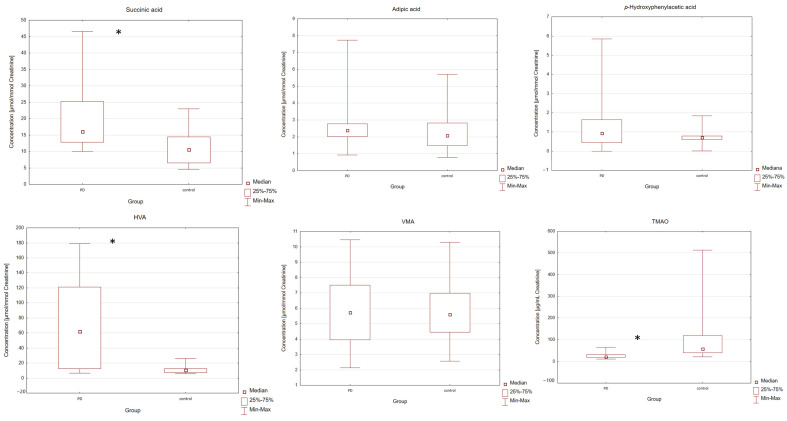
Box and Whisker plots for metabolites levels in PD and control groups. In these box plots, medians inside the 25–75% interquartile range (IQR) are presented. *—statistically significant with *p*-value < 0.05.

Validated GC-MS [24] and LC-MS methods were applied to determine the levels of succinic acids, adipic acid, *p*-hydroxyphenylacetic acid, HVA, VMA, and TMAO in the urine of PD patients and the control group. All results were calculated as a ratio of the analyte of interest and urinary creatinine concentration in units of μmol/mmol or μg/mg of creatinine. The statistical analysis of the results began by checking the distribution of the measured variables. Application of the Shapiro–Wilk test showed that the hypothesis that the data were normally distributed could be rejected [*p* < 0.05] for succinic acid, adipic acid, *p*-hydroxyphenylacetic acid, HVA, TMAO, and creatinine. A normal distribution was observed for concentration levels of VMA [*p* > 0.05]. Based on the results, individual differences in the concentrations of all metabolites between the two examined groups were found. Individual differences in the concentrations of the analyzed compounds are represented in Table 1 PD patients were found to have higher median urine levels of succinic acid, adipic acid, *p*-hydroxyphenylacetic acid, HVA, VMA and creatinine compared to the control group. In PD patients, the lower median urine levels of TMAO were also observed. The levels of analyzed compounds were compared between the two groups using a Mann–Whitney U test. The application of this test showed a significant difference between the examined groups. Differences with a *p*-value lower than 0.05 were considered significant. Individual differences in the levels of compounds between the two groups (PD and control) were found. Statistical data shows that the PD patients were characterized by a statistically significantly higher level of succinic acid [*p* = 0.0018] and HVA [*p* = 0.0002] and a statistically significantly lower level of TMAO [*p* = 1.65 × 10^−5^] in the urine of the PD group compared with the control group. For adipic acid, *p*-hydroxyphenylacetic acid, VMA and creatinine, no statistically significant difference was observed between the PD and control groups [*p* > 0.05]. The levels of VMA were compared between the two groups using an equivalent of Student’s *t*-test for unequal variances—the Cochran-Cox test (Table 1). In order to illustrate the measures of position, dispersion and asymmetry of the variables, Box and Whisker plots were included (Figure 1). The gender and age of patients in this case did not influence the concentrations of bacterial metabolites [*p* > 0.05].

In this study, seven gut microbiota-related metabolites were analyzed in urine samples from PD patients and control subjects. To address the risk of false positives due to multiple statistical tests, we applied the Benjamini–Hochberg procedure to control the false discovery rate (FDR), using a significance threshold of α = 0.05. After FDR correction, statistically significant differences remained for TMAO, HVA, and succinic acid. These findings support the robustness of the observed differences in metabolite levels between PD and control groups. Given the exploratory and pilot nature of this study, the use of FDR is particularly appropriate, as it offers a better balance between discovery sensitivity and type I error control in small-sample, biomarker-oriented research.

There were no individual differences in bacterial metabolites in subgroups of participants with PD, parkinsonian syndromes, and the control group. The group of people with parkinsonian syndromes consists of only two patients. Due to the small number of participants, further studies are required. In addition, the presence of statistically significant correlations in the patient group was investigated in terms of the H-Y disease rating scale (H-Y1-H-Y4) and disease severity (mild, moderate, advanced). For this purpose, the Kruskal–Wallis ANOVA test was used. Individual differences in the levels of succinic acid and TMAO between the H-Y disease rating scale and disease severity were found (Table 2).

**Figure 2:**
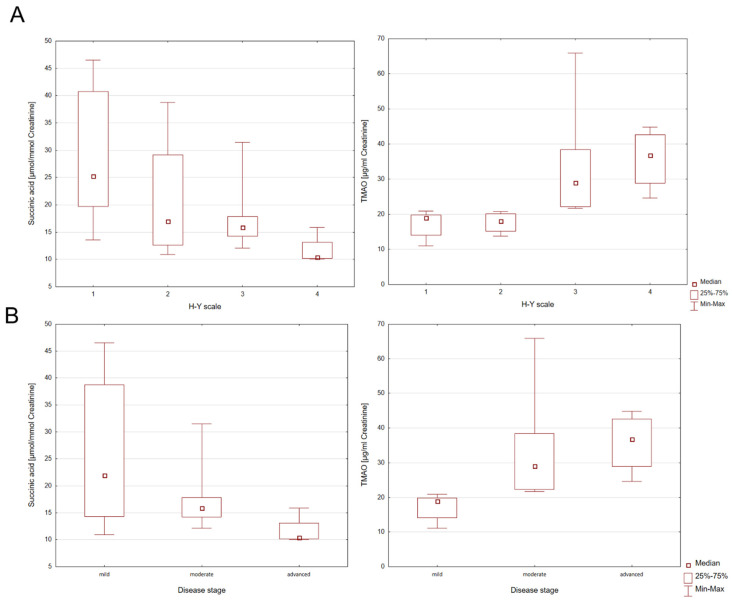
Box and Whisker plots for succinic acid and TMAO categorized in terms of (**A**) Hoehn and Yahr (H-Y) disease rating scale (H-Y1–H-Y4); (**B**) disease severity scale (mild, moderate, advanced).

Statistically significant differences for succinic acid in H-Y1 and H-Y4 PD patients [z = 2.8805; *p* = 0.0238] were observed. However, for TMAO, statistically significant differences for H-Y1 and H-Y3 [z = 2.7325; *p* = 0.0377] and H-Y1 and H-Y4 [z = 2.8804; *p* = 0.0024] were found. When considering the stage of the disease (mild, moderate, advanced), statistically significant differences for succinic acid between mild and advanced stages of PD [z = 2.6571; *p* = 0.0236] were found. In the PD group statistically significant differences were also identified for TMAO between the mild and moderate stage [z = 3.0551; *p* = 0.0067] and mild and advanced disease stages [z = 3.1428; *p* = 0.0050], which is graphically shown in the Box and Whisker plots in Figure 2.

Moreover, Spearman’s correlation coefficient for the examined compounds was calculated. This nonparametric correlation analysis allows the strength and direction of association between two ranked variables to be measured. In the PD group, the statistical analysis showed a positive correlation between levels of succinic acid and creatinine (r = 0.4571; *p* = 0.0427) and negative correlations between levels of creatinine and adipic acid (r = −0.5459; *p* = 0.0128) and HVA (r = −0.4947; *p* = 0.0266). More importantly, in the study group we also observed negative correlations between levels of succinic acid and the H-Y disease rating scale (r = −0.6307; *p* = 0.0028) and disease stage (r = −0.5801; *p* = 0.0073), and also a stronger positive correlation between levels of TMAO in the urine in PD patients the H-Y disease rating scale (r = 0.8098; *p* = 0.00001) and disease stage (r = 0.8521; *p* = 0.00002).

**Figure 3:**
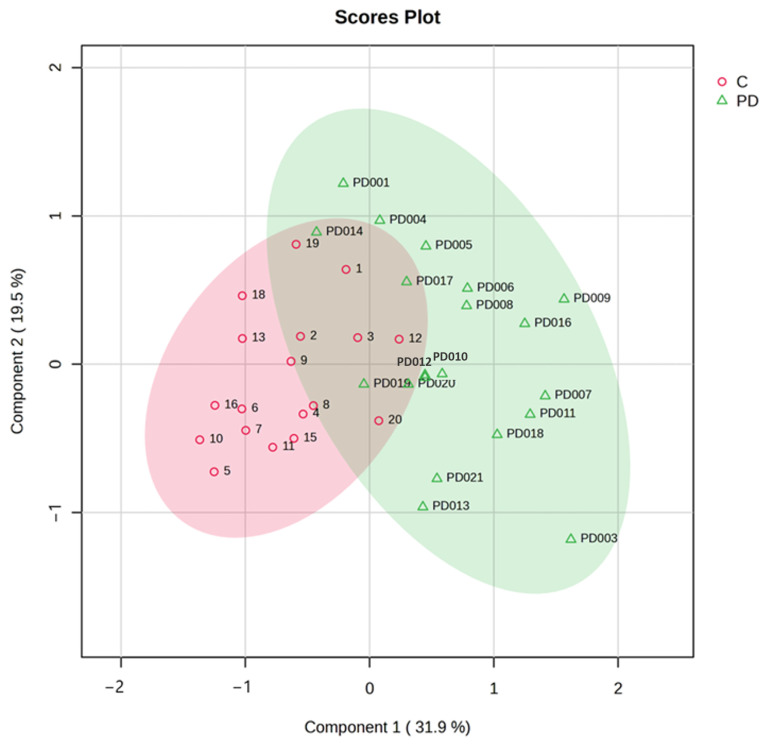
Partial least squares-discriminant analysis (PLS-DA) between PD patients and normal controls. C—control group; PD—group of people with PD.

To comprehensively evaluate metabolic patterns associated with Parkinson’s disease (PD), we employed both unsupervised and supervised multivariate approaches: principal component analysis (PCA) and partial least squares-discriminant analysis (PLS-DA). These complementary techniques provide distinct advantages for metabolomic data interpretation. PCA allowed us to visualize inherent clustering patterns in the data, identify major sources of variance among samples, detect potential outliers in the dataset and also reveal correlations between different metabolites. The PCA model incorporated all quantified metabolites (TMAO, succinic acid, HVA, and other compounds) after appropriate data scaling. Our analysis revealed a clear separation between PD and control groups along principal components 1 (PC1) and 2 (PC2), which explained 66% of the total variance. Notably, we observed a gradient along PC2 corresponding to disease severity as measured by Hoehn and Yahr staging, suggesting progressive metabolic alterations with PD advancement. Subsequently, PLS-DA, a supervised approach, was utilized to maximize the separation between predefined groups (PD vs. controls) and identify the most discriminative metabolites. The PLS-DA revealed a clear separation of samples into two distinct clusters—PD (green) and control (red)—suggesting the presence of altered or dysregulated urinary metabolites in PD patients compared to controls. The PLS-DA model demonstrated robust performance with an R^2^ of 0.641, an accuracy of 0.838, and a Q^2^ value of 0.506 across three components (Figure 3).

To evaluate the significance of the selected metabolites, the performance of the PLS-DA model was assessed in terms of prediction accuracy and permutation test. Cross-validation was employed to ensure no model overfitting, and the supervised model was also validated by performing a random permutation test (n = 1000), which produced a statistically significant result (*p* < 0.001) showing high discriminative and predictive power. These findings complement our univariate observations of PD being characterized by a distinct urinary metabolic profile and not by individual metabolite alterations. Collectively, the PCA and PLS-DA highlight the potential of urinary metabolomics for establishing multivariate panels of biomarkers for the diagnosis and monitoring of PD.ROC curve analysis was employed to assess the predictive potential of individual metabolites. To avoid overparameterization and increase model discriminative capability, we adopted a refinement strategy. This involved building a PLS-DA model on an outlier-filtered dataset followed by a second PLS-DA of the most relevant metabolites.

**Figure 4:**
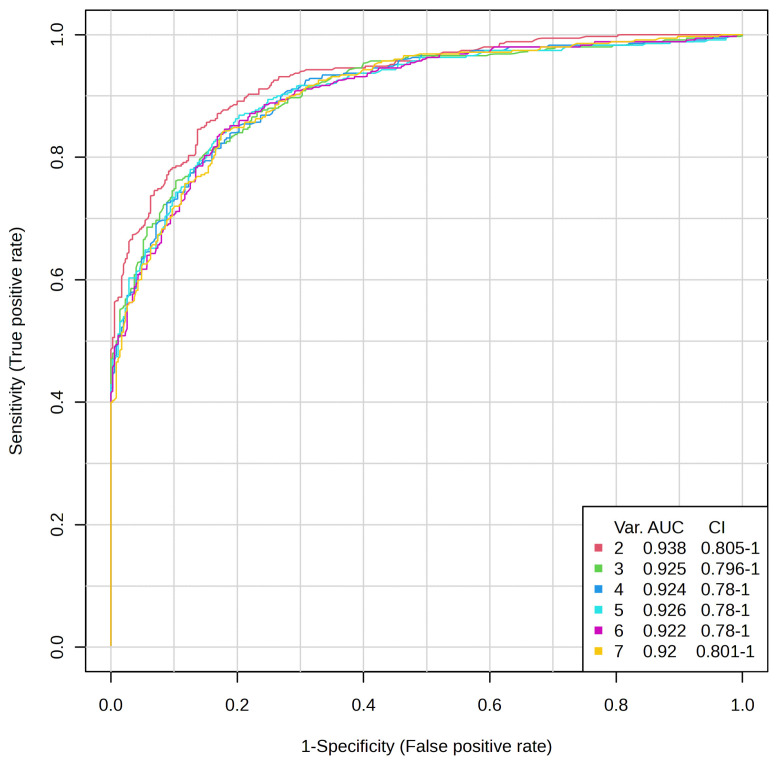
The receiver operating characteristic curve (ROC curve) analysis for the composite metabolites.

An ROC curve was produced using a *t*-test *p*-value lower than 0.05. The area under the ROC curve (AUC values) (Figure 4) was above 0.92–0.94, and we used 2–7 *t*-test metabolites to perform the biomarker analysis.

**Figure 5:**
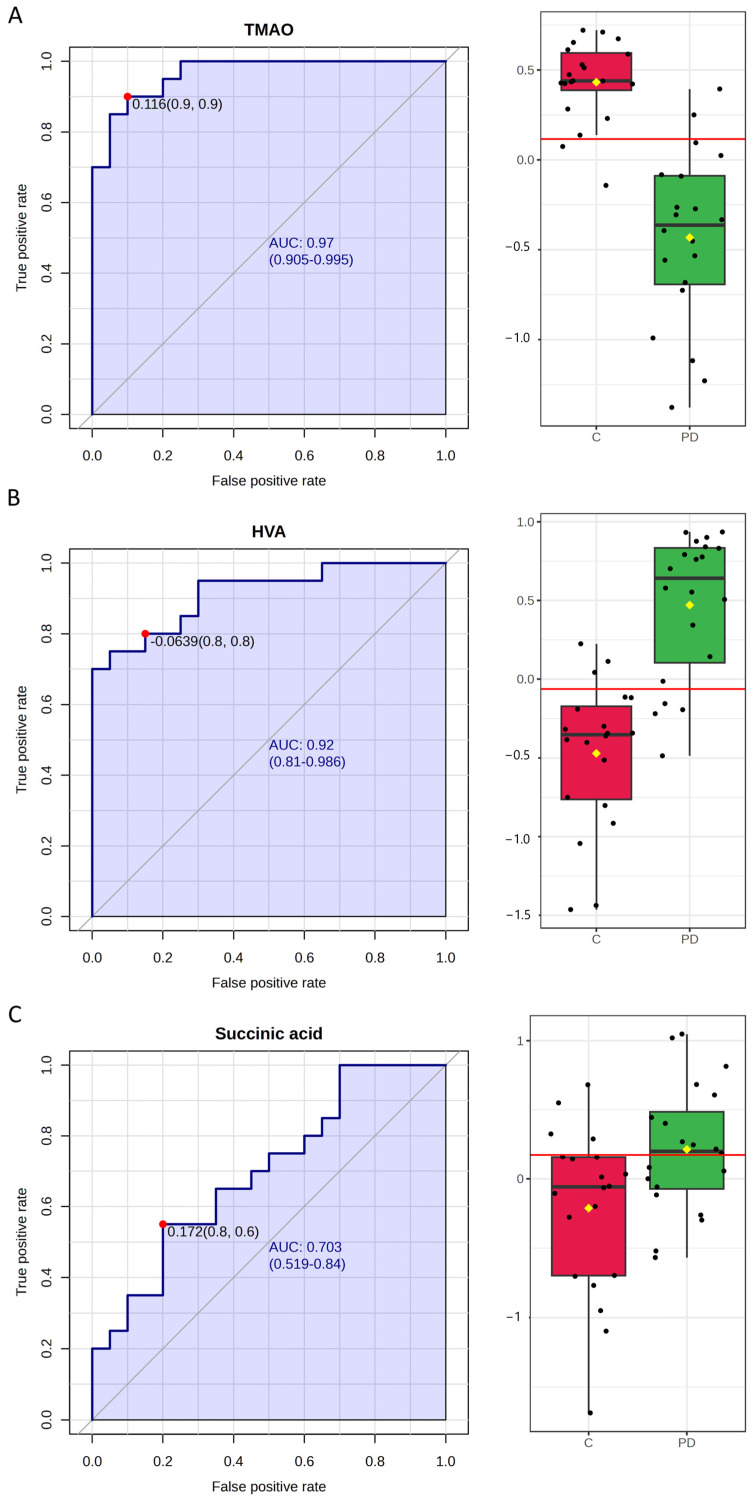
ROC curve analysis for the 3 significantly changed metabolites: (**A**) TMAO, (**B**) HVA, and (**C**) succinic acid.

Among all the metabolites, three demonstrated an adequate potential to distinguish between the PD group and controls, with an area under the ROC curve (AUC) greater than 0.7. TMAO showed the greatest AUC (0.96) for distinguishing between the PD group and controls, followed by HVA (AUC = 0.91), and succinic acid (AUC = 0.70) (Figure 5). These findings highlight the potential of leveraging a combination of identified metabolite biomarkers to develop a machine learning-based algorithm for the diagnosis of PD.

## 3. Discussion

Numerous scientific reports indicate that patients with PD have an altered gut microbiota [7,8,9,10,25,26,27,28,29,30,31,32,33]. For this reason, increasing attention is being paid to the role of gut microbiota, especially their role in neuroinflammation or neurodegeneration. To date, it is still unclear whether the gut microbiota is a trigger for PD or whether its changes are a consequence of the developing disease. Undoubtedly, the microbiota is a key contributor to neuroinflammation and neurodegeneration via the brain–gut axis. One of the ways in which the gut microbiota interacts with the brain is through the bacterial metabolites it produces, which further enter the host via the brain–gut axis [22,33,34,35,36,37,38]. The present study evaluated the levels of metabolites connected with gut microbiota, such as succinic acid, *p*-hydroxyphenylacetic acid, HVA, VMA, adipic acid, and TMAO, and also their changes in PD patients and healthy subjects. It was also examined whether there were correlations between the levels of these metabolites and the severity of the disease.

For this analysis, two complementary analytical platforms were employed: GC-MS for the detection of succinic acid, HVA, and VMA, and LC-MS/MS for the quantitation of TMAO. The selection of these methods was predicated on the physicochemical properties of the target metabolites. GC-MS, following derivatization, showed good sensitivity and chromatographic resolution for low-molecular-weight and heat-stable organic acids. Its application was most appropriate for aromatic or carboxylic-group-containing compounds, such as HVA and succinic acid, with quantitative reproducibility with low LOD. The limitation is that the method involves chemical derivatization, which can extend the sample preparation time and introduce variability if not tightly controlled. On the other hand, LC-MS/MS was selected for TMAO due to its high thermal lability and polarity that renders it unsuitable for GC-based detection without advanced derivatization. The LC-MS/MS method, especially in conjunction with hydrophilic interaction chromatography (HILIC), enabled the direct, rapid, highly selective, highly sensitive, and reproducible determination of TMAO. This also afforded minimal sample preparation as well as eliminating potential degradation of the labile species [39]. Together, GC-MS and LC-MS/MS allowed us to include a broader metabolic profile with optimal analysis performance for each class of metabolites. Such a two-platform strategy enhances the specificity and accuracy of the measurements of the metabolites and allows for their potential as strong biomarkers in the diagnosis of PD.

Our findings reveal a perturbation in bacterial metabolites in the urine of patients with PD compared to the control group. Changes were observed in the levels of succinic acid, HVA, and TMAO in the PD group. In addition, the levels of succinic acid and TMAO were associated with the stage of disease according to the Hoehn and Yahr (H-Y) disease rating scale and the degree of disease severity (mild, moderate, advanced). Hence, it is presumed that the changes observed in the levels of bacterial metabolites in the urine of patients with PD may be useful in understanding the metabolic regulation of the entire host body and the progression of neurodegeneration observed.

TMAO activates pro-inflammatory pathways, such as NF-kB signaling, causing the exacerbation of neuroinflammation. The NF-kB pathway plays a key role in the expression of pro-inflammatory genes in response to various stimuli associated with neurotransmitters. Moreover, this metabolite affects the conformation and aggregation of α-Syn in the brain, a hallmark of PD. In addition, it models the expression of various miRNAs involved in neurodegenerative processes leading to disease progression [40]. The TMAO determined in various physiological fluids has been linked to neurodegeneration and neuroinflammation processes observed in PD. However, these results are inconclusive. Altered levels of TMAO in PD patients are observed, with some studies showing an increase in this metabolite, and others showing a decrease in the various physiological fluids examined. The study conducted by Qiao et al. (2023) [41] has shown that higher levels of TMAO can induce neuroinflammation and are strongly associated with central nervous system diseases. Researchers have observed that high levels of serum TMAO are associated with worsening brain pathology by promoting striatal dopamine metabolism in acute PD model mice, resulting in increased neuroinflammation. Neuroinflammation is one of the pathological factors involved in causing PD [41]. The study conducted by Voigt et al. (2022) [1] focused on the analysis of metabolites linked to the gut microbiota in PD patients such as lactic acid, succinic acid, and TMAO. They observed that PD subjects had higher plasma levels of TMA and/or TMAO than the controls did. Available results indicate that TMAO levels are higher in both treated and untreated PD subjects compared to healthy control subjects [1]. In the literature, there are also reports of reduced TMAO levels observed in PD patients. Conversely, Chung et al. (2021) [21] observed reduced plasma TMAO levels in PD patients. They measured TMAO in 85 drug-naïve patients with an early stage of PD and 20 controls. The researchers concluded that lower TMAO levels are associated with an increased levodopa equivalent dose (LED), which is associated with an increased risk of PD conversion to dementia [21]. Reduced TMAO levels in PD patients compared to controls may be related to dysbiosis of the gut microbiota, which affects the metabolism of choline and carnitine, precursors of TMAO. The difference in the obtained results may also be related to factors specific to PD patients, such as lifestyle, diet or disease characteristics. Moreover, the reduced levels of TMAO may result in the formation of partially folded α-syn in a non-physiological state, suggesting a hormetic effect of this bacterial metabolite [21,42,43].

We also observed increased levels of bacteria-derived metabolites such as succinic acid in the urine of PD patients compared to the control group. Moreover, succinic acid levels were negatively correlated with the stage of disease according to the H-Y disease rating scale (r = −0.6307) and the degree of disease severity (r = −0.5801). The levels of bacteria-derived metabolites were also the subject of Voigt’s research [1]. He focused on the determination of short-chain and branched-chain fatty acids (lactate, isovalerate, succinate) in naïve and treated PD patients. The authors indicated a positive correlation between succinic acid and disease severity in PD patients, which remains consistent with our results. In addition, a higher ratio of TMAO to butyric acid was demonstrated in PD patients compared to controls, indicating the pro-inflammatory changes observed in the metabolite profiles of PD patients. The researchers observed that disease duration and severity as assessed by H-Y stage did not correlate with any determined bacterial metabolite. Correlations were identified between disease severity assessed by the MDS-UPDRS and levels of succinic acid, which suggested that elevated succinic acid levels correlated with the lower severity of PD symptoms [1]. Kumari et al. (2020) [44] also found significantly increased levels of succinate in the urine of PD patients in comparison with the control group and pointed out a significant association between succinate levels and UPDRS motor scores of patients with PD (r = 0.24), but did not observe any association with the disease duration [44]. Similar results were presented by Pathan et al. (2021) [45]. They observed an increased concentration of succinate and reduced concentration of formic acid in the plasma of three study groups (PD patients, patients with atypical parkinsonian disorders of multiple system atrophy (MSA), and patients with progressive supranuclear palsy (PSP)). The authors suggest that increased succinate levels affect mitochondrial dysfunction and the citric acid cycle in PD [45]. This is confirmed in the literature, as one of the substrates in the citric acid cycle is succinate. It is oxidized by succinate dehydrogenase. The oxidation process is coupled with electron transfer to form ubiquinone via the mitochondrial respiratory chain [46]. In addition, succinate participates in several key metabolic pathways and is involved in epigenetic processes and the formation and elimination of reactive oxygen species. Elevated succinate levels may indicate the activation of specific inflammatory pathways. Succinate has a signaling function in inflammatory processes through the GPR91 receptor [47]. For this reason, elevated succinate levels may be directly linked to mitochondrial dysfunction, oxidative stress and PD [46,47]. It should be noted that mitochondrial dysfunction plays a key role in the pathogenesis of PD. Through reduced ATP synthesis, defects in complex I of the mitochondrial respiratory chain occur, which may be responsible for the neurodegeneration observed in PD [45]. The change in urinary levels indicates that PD is not just a central nervous system problem—its impact extends to the metabolism of the entire body.

By contrast, the opposite results were presented by Vascellaria et al. [34], who observed lower levels of succinic acid in stool samples in the PD group, which may indicate a link between bacterial metabolites such as succinic acid and the occurrence of intestinal homeostasis disorder in PD patients [34]. Similar results were presented by Fernández-Veledo and Vendrell (2019) [22], who indicated that fecal succinic acid levels in PD patients are reduced and may be related to the severity of the disease [22].

Another important metabolite that has been determined at elevated levels in PD patients is the neurotransmitter HVA. We observed a statistically significantly higher level of HVA and no statistically significant difference in VMA in the urine of PD patients compared with the control group. HVA is a major metabolite of dopamine (DA), one of the key neurotransmitters in the brain. In the treatment of PD, drugs that increase dopamine levels such as levadopa (a dopamine precursor) are used. During a successful therapy, HVA levels increase, indicating that dopamine metabolism is returning to a more active state. Interestingly, HVA can be linked to *Roseburia intestinalis*, although they do not directly produce this metabolite, they influence the increase in the amount of resident *Bifidobacterium longum* in the intestine. It is interesting that B. longum can directly produce HVA and R. intestinalis promotes its growth [23]. The gut–brain axis may mediate how changes in the microbiota affect neurotransmitter function in the CNS.

In the scientific literature, we find manuscripts in which elevated levels of HVA were determined in the physiological fluids of patients with PD. Our findings are consistent with the previous study by Wichit et al. (2021) [48] reporting increased levels of urinary HVA in PD patients compared to controls. By contrast, VMA levels were not significantly different between the PD group and the control group [48]. Andersen et al. (2017) [49] reported increased HVA levels in the cerebrospinal fluid (CSF) of PD patients treated with L-DOPA and a decrease in untreated PD patients [49]. Stefani et al. (2017) [50] set out to investigate whether the concentration of HVA in CSF correlates with the motor impairments observed in PD patients. Patients’ motor performance was determined using the Unified Parkinson’s Disease Rating Scale-III (UPDRS-III). Patients were divided into two subgroups based on the disease stage as assessed by the Hoehn and Yahr (H-Y) scale. They observed that HVA levels increase in CSF with disease progression. The researchers observed higher HVA levels in patients with an advanced disease stage, i.e., stage > 1.5 H-Y and <2.5 H-Y, compared to PD patients with stage ≤ 1.5 H-Y. They also found that increased HVA levels are associated with the degree of motor impairment in PD patients (significant positive correlation between UPDRS-III scores and CSF HVA levels [r = 0.61; *p* < 0.0001]) [49]. However, a previous study reported a decreased level of HVA in CSF observed in patients with PD in the early stages of the disease. Moreover, there is an increase in HVA levels in CSF as the patient’s motor impairment increases, along with the progression of the disease, as confirmed by previous studies [51,52]. HVA levels reflect damage in the nigrostriatal pathway indicating a significant role of dopaminergic degeneration in the early stages of the disease, hence the observed reduced HVA levels in these early stages. Therefore, the measurement of HVA levels in CSF can be used as a biomarker of changes in disease stage to monitor the effectiveness of drug therapy used to modify the course of the disease [51,52].

Adams et al. (2023) [53] proposed a new motor classification system MDS-UPDRS TD/AR, which allows classification for disease subtyping for akinetic-rigid (AR), tremor-dominant (TD), and mixed (MX) patient subtypes. Interestingly, the researchers observed that the CSF of PD patients with the TD subtype had higher HVA levels and lower motor scores. Moreover, a specific motor subtype can be predicted based on the levels of HVA in the CSF, which is a reliable tool in selecting the disease subtype [53].

When neuronal neurodegeneration progresses in PD, the dopaminergic neurons that have not yet been affected, compensate for the loss by increasing dopamine (DA) synthesis, as well as its storage, release and turnover. This occurs through the up-regulation of aromatic amino acid decarboxylase (AADC) and vesicular monoamine transporter type 2 (VMAT2). PD patients also show increased monoamine oxidase (MAO) activity. These reactions may explain the increased levels of HVA in PD patients [54], indicating the usefulness of measuring HVA levels as a potential biomarker that indicate changes in the stage of the disease to monitor the effectiveness of the treatment used to modify the course of PD [50].

## 4. Materials and Methods

### 4.1. Subjects and Samples

Forty morning urine samples from participants were collected into sterile containers. In order to minimize the impact of diet, the urine was collected after the night. Urine samples were thoroughly mixed in order to maintain homogeneity and aliquoted. Urine was collected into 1.5 mL Eppendorf tubes (Eppendorf, Warsaw, Poland) and stored at −20 °C until chromatographic analysis. The study group (PD group) consisted of 18 subjects with idiopathic Parkinson’s disease and subjects with parkinsonian syndromes (n = 2). The inclusion criterion for the study group was a diagnosis of Parkinson’s disease or parkinsonian syndrome, made based on clinical presentation and the results of ancillary investigations, including neuroimaging of the central nervous system. The clinical diagnosis was established according to the United Kingdom Parkinson’s Disease Society Brain Bank (UKPDSBB) criteria, and the clinical condition was assessed using the Hoehn and Yahr scale [55,56]. According to Hoehn and Yahr (H-Y) staging in 1967 [56], there were 6 PD patients in stage H-Y1 and the same in H-Y3, and 4 PD patients in stage H-Y2 and H-Y4. The mean time from the onset of the disease was 4.9 ± 3.0 years (mean ± SD). PD patients were treated using L-dopa (55%, 150–900 mg/day), dopamine agonists (30%, 3–8 mg/day) and amantadine (10%, 200–300 mg/day). None of them obtained monoamine oxidase B inhibitors or catechol-O-methyl transferase inhibitors. The age range of the PD group was 47–81 years (66.2 ± 8.8). The control group consisted of 20 age- and sex-matched neurological patients, not confirmed by neuroimaging brain damage and extrapyramidal symptoms. The age range of the study participants was 39–82 years (63.3 ± 13.9). Participants with severe liver disease, renal failure, cancer, or chronic inflammatory diseases were not enrolled in this study. The demographic and clinical characteristics of patients with PD and control subjects are presented in Table 3. The study group (PD) and controls were recruited from the patients hospitalized in the Department of Neurology and Stroke, Medical University of Lodz, Poland. The study was approved by the Bioethics Committee of the Medical University of Lodz, Lodz, Poland (No. RNN/399/17/KE) and the work was conducted in accordance with the ethical guidelines of the Declaration of Helsinki.

### 4.2. Chemicals

Standard substances of heptadecanoic acid (>99%) used as an internal standard (1 mg/mL in ethyl acetate), N,O-bis(trimethylsilyl)trifluoroacetamide (BSTFA) and trimethylchlorosilane (TMCS) were obtained from Sigma-Aldrich Inc. (St. Louis, MO, USA). High-performance liquid chromatography–grade ethyl acetate, diethyl ether and chloroform were purchased from Merck (Darmstadt, Germany), and analytical-grade hydrochloric acid and sodium chloride were obtained from POCh (Gliwice, Poland). Reference standard trimethylamine N-oxide(TMAO) and internal standard TMAO-d9 were purchased from Toronto Research Chemicals (Toronto, ON, Canada).

### 4.3. Sample Preparation and Analytical Methods for Determination of Organic Acids

Organic acids (succinic acids, *p*-hydroxyphenylacetic acid, homovanillic acid (HVA), vanillylmandelic acid (VMA), adipic acid) were determined by gas chromatography coupled to mass spectrometry (GC-MS) on an Agilent 6890N Network GC system and a 5973 Network Mass Selective equipped with a capillary column (J&W Ultra Inert HP-5ms; Agilent Technology, Santa Clara, CA, USA; 30 m × 0.25 mm internal diameter; film thickness, 0.25 μm). Urine samples were mixed and aliquoted. Extraction and derivatization were performed according to a simple modification of the method described by Zhang et al. [57]. Urine samples were thawed at room temperature. Organic acids were extracted using a mixture of ethyl acetate and diethyl ether (1:1, *v*/*v*). The next step was to centrifuge the sample. The supernatant was transferred to 1.5 mL Eppendorf tubes and evaporated to dryness at room temperature. An amount of 80 µL of BSTFA/TMCS (N,O-bis[trimethylsilyl] trifluoroacetamide with trimethylchlorosilane, 100:1, *v*/*v*) was added to dry samples. Silylation reaction was continued for 10 min at 60 °C. All samples were centrifuged, transferred to glass chromatographic vials and then subjected to the GC/MS analysis. An amount of 1 μL of the supernatant was injected into the analytical column. The oven temperature was programmed initially and kept at 75 °C for 5 min and was then increased to 280 °C at 15 °C/min. The temperatures of the injector and transfer line were 250 and 280 °C, respectively. Helium was used as a carrier gas at a flow rate of 0.9 mL/min. The temperatures of the MS quadrupole and the ion source were 150 and 230 °C, respectively. Masses were acquired from *m*/*z* 50–550. Mass Hunter Workstation Software (version 10.0) was used to identify and quantify compounds. The full mass spectra were used for the structural identification and attribution of a proper retention time to each compound under analysis. The results are expressed as ratios of the urinary creatinine concentration in μmol/mmol creatinine.

### 4.4. Sample Preparation and Analytical Methods for Determination of TMAO

TMAO was determined by validated high-performance liquid chromatography coupled to mass spectrometry (LC-MS/MS) using the multiple reaction monitoring (MRM) mode on an Agilent 1260 Infinity (Agilent Technologies, Santa Clara, CA, USA) coupled to a QTRAP 4000 (AB Sciex, Framingham, MA, USA). The MRM transitions, declustering potential (DP), and collision energy (CE) were TMAO *m*/*z* 76 > 42 (DP = 66 V, CE = 53 V) and TMAO-d9 *m*/*z* 85 > 46 (DP = 61 V, CE = 59 V). Chromatographic separation was achieved using a SeQuant^®^ ZIC^®^-HILIC (50 × 2.1 mm, 5 μm, Merck) column. The column was maintained at 25 °C at a flow rate of 0.5 mL/min. The mobile phases consisted of 20 mM ammonium acetate as eluent A and acetonitrile with 0.2% formic acid as eluent B. The gradient (%B) was as follows: 0 min at 95%; 1 min at 95%; 7 min at 50%; and 8 min at 50%. The injection volume was 5 μL. Urine samples (0.1 mL) prior to injection to LC were mixed with internal standards (0.1 mL, 6 μg/mL) and acetonitrile (0.6 mL), vortexed at high speed (3 min) and centrifuged (5 min at 10,000× *g*).

### 4.5. Urinary Creatinine Determination

The level of organic acids in urine was standardized by conversion to the creatinine level. An analysis of urine for creatinine was performed by high-performance liquid chromatography according to the procedure described in detail elsewhere [58,59]. To determine the concentration of creatinine in urine samples, an Agilent 1100 series LC chromatographic system (Agilent Technologies, Waldbronn, Germany) equipped with a quaternary pump (G1310A), vacuum degasser (G1379A) and UV/VIS detector (G1314A) was used. The analyses were performed on a column (Kinetex C18; 4, 6 mm × 250 mm, 5 µm; Shim-pol). The mobile phase was phosphate buffer–acetonitrile (98:2, *v*/*v*, 15 mmol/L, pH 7.4). The flow rate was 1 mL/min, the temperature was 30 °C, and the analytical wavelength was 234 nm. Twenty microliters of a urine sample diluted 1:500 was injected into the column.

### 4.6. Methods Validation

GC-MS and LC-MS/MS methods were validated in accordance with the European Medicines Agency (EMA) guidelines for bioanalytical method validation. The GC-MS method was validated for linearity, working range, repeatability, precision, limit of detection (LOD) and limit of quantification (LOQ). The linearity of the calibration curves for all metabolites were evaluated via the R2 regression coefficients, which were in the range of 0.9957–0.9995. The measurement range was 1–60 µg/mL for succinic acid and adipic acid, and 50–500 µg/mL, 1–400 µg/mL and 10–150 µg/mL for *p*-hydroxyphenylacetic acid, HVA, and VMA, respectively. The relative standard deviations in repeatability for all metabolites ranged from 1.9 to 8.0%. Precision (CV, %) values ranged from 5.6% for succinic acid to 9.0% for HVA. The limit of detection (LOD) values were 1.9, 4.5, 46.6, 33.9, and 13.6 µg/mL for succinic acid, adipic acid, *p*-hydroxyphenylacetic acid, HVA and VMA, respectively. The limit of quantification (LOQ) values were 5.6, 13.1, 139.7, 101.8, and 40.9 µg/mL for succinic acid, adipic acid, *p*-hydroxyphenylacetic acid, HVA and VMA, respectively. The presented values indicate satisfactory sensitivity of the developed GC-MS method for organic acids in human urine. The LC-MS/MS was validated against the following parameters: the calibration curve covered a range of 10–200 µg/mg of creatinine (r > 0.998) using a 1/x weighting factor. Accuracy and precision were assessed at three concentration levels, with the accuracy ranging from 89.0 to 104.1% and the precision ranging between 2.3 and 3.9%. At the lowest limit of quantitation, accuracy and precision were 96.3 and 2.3%, respectively. The relative matrix effect was below 15%.

### 4.7. Statistical Analysis

Data were statistically evaluated using the statistical analysis package (StatSoft, Polska STATISTICA, version 13.0., Quest Software, Aliso Viejo, CA, USA). The statistical analysis began with an examination of the distributions of quantitative variables. For this purpose, basic descriptive statistics were calculated and normality was determined using the Shapiro–Wilk’s test. Student’s *t*-test and the nonparametric Mann–Whitney U test were used, as appropriate, to compare the concentration of compounds between the studied groups. All comparisons were two-sided with a *p*-value of less than 0.05 used to indicate statistical significance. Subsequently, the strength of associations between variables was measured using Spearman’s rank correlation. Metabolomics data analysis was carried out in a web-based comprehensive metabolomics data processing tool, MetaboAnalyst 6.0, available at http://www.metaboanalyst.ca (accessed on 12 April 2025).

### 4.8. Limitations

This study, however, has several important limitations. The limited sample size (n = 40), including only 20 patients with PD—of whom just two were diagnosed with atypical parkinsonian syndromes—significantly restricts the generalizability of the findings and limits the possibility of conducting meaningful subgroup analyses. Moreover, the exploratory, pilot nature of the study means it was designed primarily to generate hypotheses rather than to confirm established associations. The statistical power may also have been insufficient (power < 0.80) in detecting subtle differences in metabolite levels between the groups.

Dietary and lifestyle factors, which are known to strongly influence TMAO and related metabolites (e.g., intake of choline or L-carnitine), were not accounted for. Variables such as diet, supplementation, physical activity, and smoking status were not assessed and may have confounded the results. Additionally, the cross-sectional design, with urine samples collected at a single time point, precludes the analysis of longitudinal changes in metabolite levels and limits causal interpretations. It is also possible that random or unmeasured factors contributed to the observed metabolic differences between groups.

Despite these limitations, our study successfully captures key metabolic features detectable in the urine of PD patients. Future research involving larger, more diverse cohorts—stratified by disease severity—and including comparison groups with other neurodegenerative disorders (e.g., multiple system atrophy [MSA], progressive supranuclear palsy [PSP]) may provide more definitive insights. Moreover, parallel analyses of other biological fluids such as plasma, serum, or cerebrospinal fluid, combined with detailed dietary and lifestyle assessments, could help validate and refine the utility of these metabolic biomarkers.

## 5. Conclusions

The results of our study of the levels of bacterial metabolites in the urine of PD patients and controls shed light on potential biomarkers of disease progression. Our study showed changes in the levels of gut microbiota-associated metabolites in the urine in patients with PD. This study found that patients with PD had higher levels of succinic acid and HVA and lower levels of TMAO in their urine compared to the control group. The change in urinary succinate levels indicates that PD is not just a central nervous system problem—its impact extends to the metabolism of the entire body. Analysis of this metabolite in urine is of particular interest because it is a non-invasive method that can provide valuable information about the patient’s condition. Moreover, a correlation was also demonstrated between succinic acid and TMAO levels and the stage of disease progression, both in terms of the H-Y disease assessment scale and disease severity. Statistical analysis, based on nonparametric tests and correlations, identified several significant differences and correlations that may be important for early diagnosis, monitoring disease progression, and understanding its pathogenesis. The obtained information contributes to expanding knowledge about the dysfunction of gut microbiota-associated metabolites in PD. This emphasizes the extremely important role of monitoring the status of these metabolites in patients during the course of the disease, predicting its development and symptoms, planning optimal and individualized treatment, and monitoring the effectiveness of treatment. Further studies are necessary to better understand the role of the microbiota metabolites in the pathogenesis of PD and their potential use as diagnostic and prognostic biomarkers.

## Figures and Tables

**Table 1:** Values obtained for determined compounds in the urine samples of the entire tested population.

Metabolites	Units	Group	Median ± SE	Min–Max	Q25	Q75	SD	*p* *	*p* **
Succinic acid	µmol/mmol Creatinine	PD	14.27 ± 2.69	10.00–46.57	12.84	25.24	10.92	2.89 × 10^−3^	0.0018
		Control	10.57 ± 1.24	4.60–22.96	6.51	14.42	5.54		
Adipic acid		PD	2.38 ± 0.35	0.92–7.73	2.01	2.78	1.58	6.63 × 10^−4^	0.3648
		Control	2.08 ± 0.28	0.78–5.71	1.47	2.83	1.27		
*p*-Hydroxyphenylacetic acid		PD	0.93 ± 0.33	0.0006–5.85	0.45	1.63	1.45	5.04 × 10^−4^	0.3085
		Control	0.71 ± 0.09	0.01–1.84	0.60	0.78	0.39		
Homovanillic acid		PD	62.18 ± 12.50	6.45–179.21	12.33	121.09	55.89	4.80 × 10^−2^	0.0002
		Control	10.37 ± 0.98	5.78–25.84	7.43	12.39	4.40		
Vanillylmandelic acid		PD	5.72 ± 0.49	2.14–10.47	3.96	7.50	2.29	0.7674	0.6058
		Control	5.59 ± 0.43	2.57–10.29	4.44	6.99	1.94		
TMAO	µg/mg Creatinine	PD	21.31 ± 2.92	11.07–65.92	18.93	31.59	13.08	4.29 × 10^−3^	1.60 × 10^−5^
		Control	57.67 ± 27.96	20.79–513.40	40.73	119.71	125.03		
Creatinine	mg/mL	PD	1.05 ± 0.18	0.25–2.60	0.64	2.11	0.81	0.0275	0.5249
		Control	0.92 ± 0.15	0.07–2.53	0.63	1.73	0.69		

SE—standard error; Q25—lower quartile; Q75—upper quartile; SD—standard deviation; *p* *—*p* values calculated by a Shapiro–Wilk test, *p* **—*p* values calculated by Mann–Whitney U test.

**Table 2:** Mean and SD of urinary concentrations for the statistically significant variables in different H–Y stages of PD ranging from 1 to 4 and disease duration from mild to moderate to advanced.

	Mean	SD	Mean	SD
	Succinic Acid [µmol/mmol Creatinine]		TMAO [µg/mL Creatinine]	
H-Y disease rating scale				
H-Y1	28.51	12.65	17.29	3.84
H-Y2	20.90	12.42	17.65	3.11
H-Y3	17.90	6.92	34.39	16.60
H-Y4	11.65	2.81	35.75	8.84
Disease duration				
mild	25.47	12.48	17.43	3.39
moderate	17.90	6.92	34.39	16.60
advanced	11.65	2.81	35.75	8.84

**Table 3:** Demographic and clinical characteristics of patients with Parkinson’s disease and control subjects.

	PD Patients	Controls
Age [years]	66.2 ± 8.8	63.3 ± 13.9
Gender [%]		
Male	60%	60%
Female	40%	40%
Body Mass Index (BMI) [%]		
normal weight	45%	40%
overweight	40%	30%
obesity class I	5%	20%
obesity class II	10%	10%
Disease duration [years]	4.9 ± 3.0	-
Hoehn and Yahr (H-Y) disease rating scale	2.4 ± 3.0	-
H-Y 1	30%	
H-Y 2	20%	
H-Y 3	30%	
H-Y 4	20%	

## Data Availability

The original contributions presented in this study are included in the article. Further inquiries can be directed to the corresponding authors.

## Abbreviations

The following abbreviations are used in this manuscript:

TMAO	Trimethylamine N-oxide
HVA	Homovanillic acid
VMA	Vanillylmandelic acid
PD	Parkinson’s disease
GC-MS	Gas Chromatography–Mass Spectrometry
LC-MS/MS	Liquid Chromatography–Tandem Mass Spectrometry
H-Y	Hoehn and Yahr disease rating scale
NF-kB	Nuclear factor kappa-light-chain-enhancer of activated B cells
α-syn	α-synuclein
SCFAS	Short chain fatty acids
BCFAS	Branched chain fatty acids
TCA	Tricarboxylic acid cycle
CNS	Central nervous system
LED	Levodopa equivalent dose
MDS-UPDRS	Movement Disorder Society–Unified Parkinson’s Disease Rating Scale
MSA	Multiple system atrophy
PSP	Progressive supranuclear palsy
DA	Dopamine
CSF	Cerebrospinal fluid
AR	Akinetic-rigid
TD	Tremor-dominant
MX	Mixed patient subtypes
AADC	Aromatic amino acid decarboxylase
VMAT2	Vesicular monoamine transporter type 2
MAO	Monoamine oxidase
BMI	Body mass index
BSTFA	N,O-bis(trimethylsilyl)trifluoroacetamide
TMCS	Trimethylchlorosilane
MRM	Multiple reaction monitoring
DP	Declustering Potential
CE	Collision Energy
LOD	Limit of detection
LOQ	Limit of quantification
PCA	Principal Component Analysis
PLS-DA	Partial Least Squares Discriminant Analysis
ROC	Receiver Operating Characteristic
R^2^	Coefficient of Determination
MSA	Multiple System Atrophy
PSP	Progressive Supranuclear Palsy
DA	Dopamine
